# Visual Alignment Method for Hoisting Prefabricated Segmented Beams

**DOI:** 10.3390/s26113426

**Published:** 2026-05-28

**Authors:** Lin Xiao, Chengli Zhao

**Affiliations:** 1 CCCC Second Harbor Engineering Company Ltd., Wuhan 430040, China; 2 School of Transportation and Logistics Engineering, Wuhan University of Technology, Wuhan 430063, China

**Keywords:** prefabricated segmented beams, binocular vision, rod and hole alignment, epipolar geometry

## Abstract

During the hoisting of prefabricated segmented beams, the alignment of rods and holes mainly relies on manual operation, which suffers from low safety and efficiency. To improve the safety and efficiency of rod–hole alignment, this paper proposes a vision-based alignment method for hoisting prefabricated segmented beams. The method uses binocular vision to measure the spatial coordinates of key points on rods and holes, establishes a mathematical model for alignment, and calculates the center distance and relative rotation angle between them. An experimental platform is built and tests are conducted. The results show that the proposed method can effectively measure the center distance and rotation angle, improve measurement efficiency and safety, achieve high accuracy, and possess high practical engineering value.

## 1. Introduction

In the field of modern bridge construction, segmented beam assemblies, as an efficient, high-quality and environmentally friendly construction method, are increasingly being widely applied [1]. This construction technique divides a bridge into several sections, which are prefabricated in factories or prefabrication sites and then transported to the construction site for assembly. This significantly shortens the on-site construction period, reduces the impact on the surrounding environment, and simultaneously enhances the construction quality and structural stability of the bridge. This method is widely used in bridge construction projects, such as urban bridges, highway bridges, and cross-sea bridges. For example, in urban elevated bridge construction, segmented beam assemblies enable rapid construction in confined urban spaces and minimize traffic disruption. In the construction of large-scale cross-river and cross-sea bridges, they help improve construction efficiency and reduce construction risks [2].

During the assembly process of segmented beams, the alignment of rods and holes is a crucial step, directly affecting the stability and safety of the entire bridge structure. If the rods are not accurately aligned with the holes, this may lead to uneven force distribution on the rod and local stress concentration, thereby affecting the service life of the rod and even causing serious problems, such as deformation and cracking of the bridge structure, threatening the operational safety of the bridge. The traditional methods for aligning rods and holes, such as manual measurement and adjustment, are not only inefficient but also have difficulties in guaranteeing accuracy. With the continuous expansion of bridge construction scales and the improvement of technical standards, in-depth research on the alignment methods of rods and holes has become an important issue that urgently needs to be addressed in the field of bridge construction. In the face of complex construction environments and high-precision bridge construction requirements, the exploration of more efficient, precise, and intelligent alignment technologies is needed. This has practical significance for promoting the advancement of bridge construction technology and ensuring the quality of bridge engineering [3].

**Figure 1:**
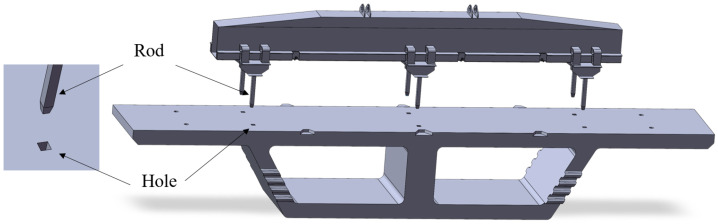
The hoisting of segmented beams.

A schematic diagram of the hoisting of segmented beams is shown in Figure 1. There are six square rods on the lifting gear, and corresponding square holes are provided on the segmented beams. After the precise alignment of hole positions with the rod is completed, the control system will insert the rod into the hole and fix them. The cross-sections of both the rod and the hole are square structures. To ensure that the square rod can be accurately embedded into the square hole, the side length of the hole is usually 10 mm longer than that of the rod.

In the early stage of the development of segmented beam assembly technology, the alignment of rods and holes mainly relied on manual operation. In this way, construction workers first used their experience and simple measuring tools to manually adjust the spacing between the rods to roughly match the spacing of the holes on the segmented beams. In actual operation, workers stood on the top surface of the segmented beams or used simple climbing equipment to manually push the rods closer to the holes. Through continuous observation and fine tuning, the rods were gradually aligned with the holes. When the rods approached the holes, workers needed to adjust the angle and position more carefully to ensure that the rods could be accurately inserted into the holes. After the alignment was completed, fastening anchors were manually installed to fix and connect the rods to the segmented beams.

This manual alignment method has many drawbacks. In terms of efficiency, the entire alignment process requires the collaborative operation of multiple workers, and each adjustment is time and labor consuming, resulting in slow assembly progress of the segmented beams. In some early bridge segmented beams assembly projects, it might take several hours or even longer to align just one set of rods and holes, seriously affecting the overall progress of the project.

In terms of safety, workers are required to operate at high altitudes and in narrow working spaces; during the operation process, they also need to frequently move and adjust the rods. Thus, there exist significant safety risks. For instance, workers may encounter accidents due to accidental falls or being hit by the rods.

As for alignment accuracy, manual operation is greatly influenced by factors such as workers’ experience, skill level, and fatigue, making it difficult to ensure that each alignment meets the high-precision requirements. This can easily lead to certain deviations between rods and holes, thereby affecting the installation quality of the segmented beams and the stability of the bridge structure. If there is a deviation in the alignment of rods and holes, during the subsequent use of the bridge, there will be uneven local force distribution on the rods, resulting in varying degrees of wear and deformation of the rods, which increases the maintenance cost and safety hazards of the bridge [4,5].

With the continuous development of bridge construction technologies and the increasing demand for construction efficiency, semi-automatic auxiliary alignment technology has gradually emerged on the basis of manual alignment. This technology introduces some simple mechanical equipment to assist construction workers in aligning rods and holes. Among them, the simple sliding device is one of the more common auxiliary devices. It is usually composed of a track, a slider and a drive device. The track is installed on the segmented beams or lifting gear, the slider can slide freely on the track, and the rods are installed on the slider. Through the drive device, such as a small motor or hydraulic device, the slider can move on the track, thereby driving the hanger to adjust its position in the horizontal direction. The construction workers only need to control the drive device to conveniently adjust the position of the rods to approach the holes and then manual fine tune to complete the final alignment [6,7].

Compared with the early pure manual alignment method, semi-automatic auxiliary alignment technology has made remarkable progress. In terms of efficiency, thanks to mechanical equipment, the position adjustment of rods is more rapid and convenient, significantly reducing the time required for alignment. Taking a real project as an example, after adopting semi-automatic auxiliary alignment technology, the alignment time for each group of rods and holes is shortened from several hours to tens of minutes, which improves construction efficiency several times. In terms of labor intensity, the workload of workers manually pushing rods has been reduced, alleviating their labor intensity and enabling them to operate in a relatively relaxed environment. However, semi-automatic auxiliary alignment technology also has certain limitations. In terms of alignment accuracy, although it has been improved compared with pure manual methods, it is still difficult to meet the requirements of some high-precision bridge construction projects. In particular, when dealing with complex segmented beam structures and strict design standards, certain deviations may still occur. Semi-automatic auxiliary alignment technology relies to a certain extent on the normal operation of mechanical equipment. If the equipment malfunctions, it may lead to construction interruption and affect the project progress [8]. In recent years, with the rapid development of advanced sensor technology and automatic control technology, intelligent and automatic alignment methods have gradually emerged in the assembly of segmented beams and have been widely applied [9,10,11,12,13]. These methods utilize advanced sensors, such as laser sensors and vision sensors, to obtain the position information of rods and holes in real time. For instance, by leveraging machine vision recognition technology, the position, shape and posture of the holes can be identified and analyzed through cameras installed on the lifting gear or segmented beams, and the obtained image information is transmitted to the control system. The control system processes and analyzes the data collected by the sensors based on advanced algorithms, such as pattern recognition algorithms and image processing algorithms [14,15,16]. The position deviation and angle deviation between rods and holes are calculated using these algorithms. Based on the calculation results, the automatic control actuators, such as electric push rods and hydraulic cylinders, precisely adjust the position and angle of the rods to achieve automatic alignment between rods and holes. In some large-scale bridge construction projects, intelligent and automated alignment systems can complete high-precision alignment of rods and holes in a short time, greatly improving construction efficiency and quality.

Intelligent and automated alignment methods have many significant advantages. In terms of accuracy, they can achieve millimeter-level or even higher precision alignment, effectively ensuring the installation quality of the segmented beams, reducing structural hazards caused by alignment deviations, and enhancing the overall stability and safety of the bridge. In terms of efficiency, the automated operation process significantly reduces the alignment time. Compared with traditional manual or semi-automatic alignment methods, the efficiency is increased several times or even dozens of times, thus meeting the demands of large-scale and high-efficiency bridge construction. From the perspective of safety guarantee, these methods reduce the working time and workload of workers in high-altitude and dangerous environments, lower construction safety risks, and ensure the personal safety of construction personnel. Intelligent and automated alignment methods can also achieve real-time monitoring and recording of data, facilitating quality traceability and management of the construction process, and providing strong support for the intelligent and digital development of bridge construction.

Traditional rod–hole alignment relies on manual naked-eye observation or monocular vision, which has obvious accuracy defects. Manual alignment is easily affected by occlusion and experience differences. Monocular vision can only obtain two-dimensional plane information and cannot determine the depth deviation between rods and holes. The three-dimensional positioning capability of binocular vision can better solve this problem [17]. In rod–hole alignment, the binocular vision method can output three-dimensional deviation values in real time, guiding actuators to make precise adjustments. This avoids problems such as the shear deformation of rods and stress concentration at the edge of holes caused by alignment deviation, and it significantly reduces long-term maintenance costs.

To meet the demand for the intelligent and digital development of precast segmented beam assembly, upgrade the unmanned and intelligent level of beam hoisting and lowering, and enhance operational safety, this paper proposes a binocular vision-based hoisting alignment method for precast segmented beams. In response to the problems of high dependence on manual operation, low automation, insufficient alignment accuracy, and significant safety risks in traditional segmented beam hoisting operations, the proposed method uses binocular vision to realize the spatial perception of key components. By acquiring the spatial coordinates of the matching holes and rods in real time, it establishes a mathematical model for attitude analysis and calculates the center distance and relative rotation angle between rods and holes. This method effectively improves the precision, efficiency, and safety of the alignment process, reduces human errors and construction risks, and provides technical support for the intelligent and unmanned construction of segmented beam assembly.

This paper has the following innovative points: (1) A stereo camera calibration method based on constraint conditions is proposed. (2) It presents the calculation methods for the center distance and rotation angle in rod–hole alignment. (3) A stereo camera layout method based on the vision-based process of aligning rods and holes is proposed. The organization of this paper is as follows: Section 1 introduces the current research status of rod–hole alignment methods. Section 2 introduces the visual measurement methods for holes and rods. Section 3 establishes an engineering experimental platform and presents the experiments. Section 4 summarizes the full paper.

## 2. Method

### 2.1. Calibration Method for Stereo Cameras Based on Constraint Conditions

**Figure 2:**
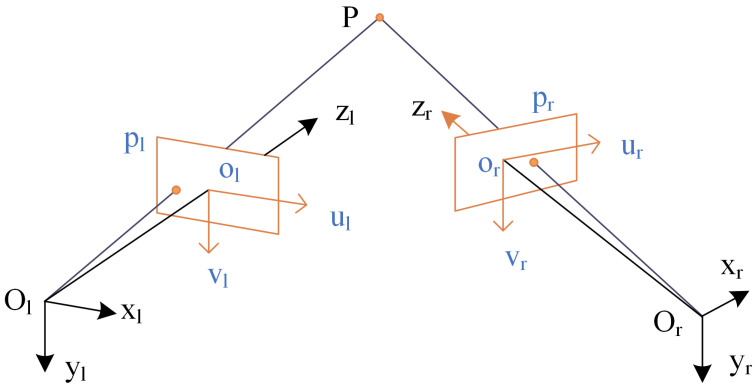
Binocular vision principle.

To obtain the three-dimensional data of the object to be measured, a binocular vision measurement model [18,19] is required. As shown in Figure 2, the principle of binocular vision measurement is illustrated. Point P is a spatial point, and the projection points of P on the two images are $p_{r}$ and $p_{l}$, respectively. Theoretically, points P, $p_{l}$, and $O_{l}$ are collinear, and points P, $p_{r}$, and $O_{r}$ are also collinear.

The projections of point P on left image and right image are $p_{l}$ and $p_{r}$, respectively. Points P, $p_{l}$ and $p_{r}$ satisfy the following relationship:

(1) $$\left\{\begin{array}{c} P_{l}\;=\;R_{l}P_{w}\;+\;t_{l}\\ P_{r}\;=\;R_{r}P_{w}\;+\;t_{r} \end{array}\right.$$

Among them, $R_{l}$ is the rotation matrix of the left camera, $t_{l}$ is the translation matrix of the left camera, $R_{r}$ is the rotation matrix of the right camera, and $t_{r}$ is the translation matrix of the right camera.

The point P in the left and right camera coordinate systems can be associated by the rotation matrix and the translation matrix, and the calculation formulas are as follows:

(2) $$P_{l}=R_{d}\left(P_{r}-t_{d}\right)$$

Among them, $R_{d}$ and $t_{d}$ are the rotation matrix and translation matrix between the left and right cameras, respectively. Therefore, the following relationship can be derived:

(3) $$\left\{\begin{array}{c} R_{d}\;=\;R_{r}(R_{l})t_{d}\\ t_{d}\;=\;t_{t}-R_{d}t_{l} \end{array}\right.$$

The binocular vision measurement system uses two cameras to simulate the positioning function of the human eye, and it determines the three-dimensional coordinates of spatial points by the spatial posture of the cameras and image points. Generally speaking, for the convenience of calculation, the world coordinate system is superimposed with one of the camera coordinate systems. The projection matrices of the left and right cameras are as follows, respectively:

(4) $$\begin{array}{c} M_{L}\;=\;K_{L}\left[\begin{array}{cc} I & 0 \end{array}\right]\\ M_{R}\;=\;K_{R}\left[\begin{array}{cc} R & t \end{array}\right] \end{array}$$

Among them, $K_{L}$ and $K_{R}$ are the left and right camera intrinsic parameter matrices, respectively, and *I* is a 3 × 3 identity matrix. After obtaining the intrinsic and extrinsic parameters of the two cameras through the calibration method, the three-dimensional coordinates of the corresponding spatial points can be calculated based on the corresponding image points on the left and right images.

With the above equations, the relationship between the left and right cameras can be obtained as follows.

(5) $$s_{r}\left[\begin{array}{c} u_{r}\\ v_{r}\\ 1 \end{array}\right]=\left[\begin{array}{cccc} f_{r}r_{1} & f_{r}r_{2} & f_{r}r_{3} & f_{r}t_{x}\\ f_{r}r_{4} & f_{r}r_{5} & f_{r}r_{6} & f_{r}t_{y}\\ r_{7} & r_{8} & r_{9} & t_{z} \end{array}\right]\left[\begin{array}{c} zu_{l}/f_{l}\\ zv_{l}/f_{l}\\ z\\ 1 \end{array}\right]$$

Through Equation (5) and Figure 2, the space coordinate of point *P* can be expressed by the following equation.

(6) $$x=zu_{l}/f_{l}$$

(7) $$y=zv_{l}/f_{l}$$

(8) $$\begin{array}{c} z=\frac{f_{l}\left(f_{r}t_{x}-u_{r}t_{z}\right)}{u_{r}\left(r_{7}u_{l}+r_{8}v_{l}+f_{l}r_{9}\right)-f_{r}\left(r_{1}u_{l}+r_{2}v_{l}+r_{3}f_{l}\right)}\\ =\frac{f_{l}\left(f_{r}t_{y}-v_{r}t_{z}\right)}{v_{r}\left(r_{7}u_{l}+r_{8}v_{l}+f_{l}r_{9}\right)-f_{r}\left(r_{4}u_{l}+r_{5}v_{l}+r_{6}f_{l}\right)} \end{array}$$

Among them, $f_{l}$ and $f_{r}$ are the scale factors of the left and right cameras; $r_{1}$–$r_{9}$ are the coefficients. Since $t_{x}$ is not equal to 0, $T^{\prime }={\left(1,t_{y}^{\prime },t_{z}^{\prime }\right)}^{T}$, so Equations (6)–(8) can be expressed as f(x)=0. The specific equation is as follows.

(9) $$\begin{array}{c} \left(f_{r}-u_{r}tl_{z}\right)\left(r_{4}u_{l}+r_{5}v_{l}+f_{l}r_{6}\right)-\left(f_{r}t_{y}^{\prime }-v_{r}tl_{z}\right)\left(r_{1}u_{l}+r_{2}v_{l}+f_{l}r_{3}\right)\\ -\left(v_{r}-u_{r}tl_{y}\right)\left(r_{7}u_{l}+r_{8}v_{l}+f_{l}r_{9}\right)=0 \end{array}$$

(10) $$x={\left(t_{y}^{\prime },t_{z}^{\prime },r_{1},r_{2},r_{3},r_{4},r_{5},r_{6},r_{7},r_{8},r_{9}\right)}^{T}$$

The length of the reference line can be calculated from the following equation.

(11) $$f_{l}^{2}L^{2}={\left(z_{1}u_{l1}-z_{49}u_{l49}\right)}^{2}+{\left(z_{1}v_{l1}-z_{49}v_{l49}\right)}^{2}+f_{l}^{2}{\left(z_{1}-z_{49}\right)}^{2}$$

If the calibration board has multiple reference lines, there is the following equation, whose lengths are $L_{i}$. To minimize the overall error of the measurement, the constraints of reference lines need to be comprehensively considered, so the translation vector *T* is as follows.

(12) $$T=T^{*}/L^{*}\left(\sum_{1}^{n}L_{i}\;\right)/n$$

Among them, *n* represents the number of reference lines.

### 2.2. Epipolar Geometric Search

**Figure 3:**
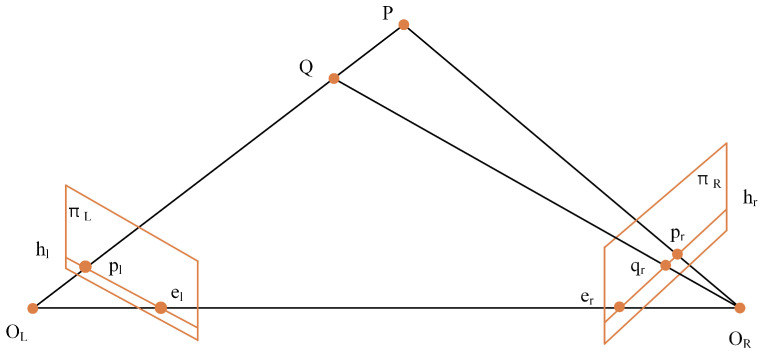
Epipolar search principle.

The core idea of standard binocular system measurement is to rectify the left and right images using the calibration parameters of the two cameras and then calculate the depth information of the object to be measured via geometric relationships [20,21,22]. Figure 3 shows the correction schematic of the binocular system. Points $O_{L}$ and $O_{R}$ are the projection centers of the cameras, and the line connecting them $O_{L}O_{R}$ is the baseline. P represents the spatial point to be measured, and its projections on the images are $P_{l}$ and $P_{r}$, respectively. The planes of the left and right images are denoted as plane ${ß}_{L}$ and plane ${ß}_{R}$, respectively. The intersection lines $h_{l}e_{l}$ and $h_{r}e_{r}$ between the epipolar plane ${\mathrm{PO}}_{L}O_{R}$ and the planes ${ß}_{L}$ and ${ß}_{R}$ are called epipolar lines. Point Q lies on the line $O_{L}P$, and its projection on plane ${ß}_{L}$ is also $P_{l}$. The projection of point P onto plane ${ß}_{R}$ must lie on the line $h_{r}e_{r}$. This is the epipolar constraint, which forms the basis of stereo matching. The epipolar constraint restricts the search and matching of pixels to a certain line segment in the image, thus reducing the computational load of pixel matching. By using the epipolar constraint, the corresponding points can be quickly located in the other image.

According to the geometric relationship, there are the following coplanar equations on the epipolar plane:

(13) $$\left|\begin{array}{ccc} B_{x} & B_{y} & B_{z}\\ x_{l} & y_{l} & -f\\ x_{e_{1}} & y_{e_{1}} & -f \end{array}\right|=0$$

Among them, $B_{x}$, $B_{y}$ and $B_{z}$ are the baseline components; $x_{l}$ and $y_{l}$ are the image plane coordinates of point $P_{l}$ which are known; and *f* is the focal length of the camera. The relationship between $x_{l}$ and $y_{l}$ is as follows:

(14) $$y_{l}=\left(A/B\right)\cdot x_{l}+\left(C/B\right)\cdot f$$

Among them,

(15) $$\left\{\begin{array}{c} A=f\cdot B_{l}+y_{l}\cdot B_{z}\\ B=f\cdot B_{l}+x_{l}\cdot B_{z}\\ C=y_{l}\cdot B_{x}-x_{l}\cdot B_{y} \end{array}\right.$$

For the epipolar line of the right image, the coplanar conditional equation can also be used to solve it:

(16) $$\left|\begin{array}{ccc} -Bx^{\prime } & -By^{\prime } & -Bz^{\prime }\\ x_{r} & y_{r} & -f^{\prime }\\ x_{e_{2}} & y_{e_{2}} & -f \end{array}\right|=0$$

Among them,

(17) $$\left\{\begin{array}{c} {}[\;B_{x}^{\prime }\;\;B_{y}^{\prime }\;\;B_{z}^{\prime }\;]=[\;B_{x}\;\;B_{y}\;\;B_{z}\;]M_{r}\\ {}[\;x_{r}\;\;y_{r}\;\;f\;]=[\;x_{e_{2}}\;\;y_{e_{2}}\;\;f\;]M_{r} \end{array}\right.$$

$M_{r}$ is the rotation matrix between the left image space auxiliary coordinate system and the right image space auxiliary coordinate system. *x* and *y* are the image plane coordinates of point $P_{r}$, which are known. The relationship between *x* and *y* is as follows:

(18) $$y_{r}=\left(A^{\prime }/B^{\prime }\right)\cdot x_{r}+\left(C^{\prime }/B^{\prime }\right)\cdot f$$

Among them,

(19) $$\left\{\begin{array}{c} A^{\prime }=f\cdot B_{x}^{\prime }+y_{r}\cdot B_{z}^{\prime }\\ B^{\prime }=f\cdot B_{x}^{\prime }+x_{r}\cdot B_{z}^{\prime }\\ C^{\prime }=y_{r}\cdot B_{x}^{\prime }-x_{r}\cdot B_{y}^{\prime } \end{array}\right.$$

Therefore, the relationship between *x* and *y* can be obtained, and the epipolar equation on the right image is acquired.

**Figure 4:**
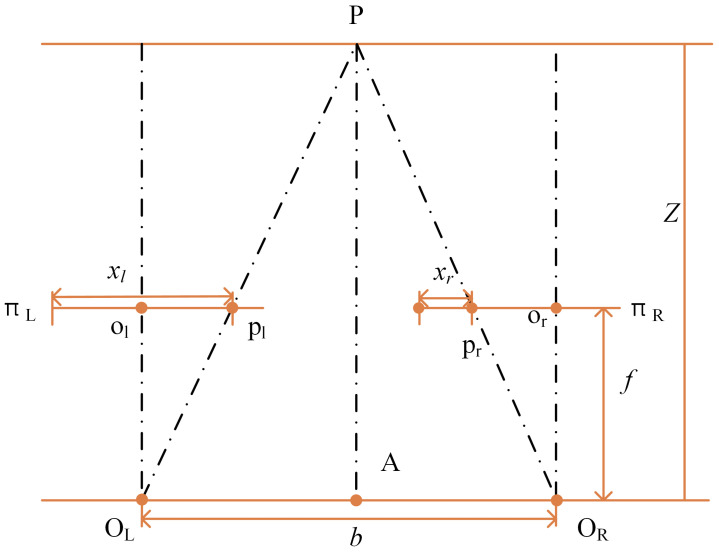
Disparity calculation principle.

As shown in Figure 4, after the stereo camera is calibrated and its parameters are known, stereo correction can be performed using the epipolar constraint so that the optical axes of the left and right cameras are parallel, which means the image planes are parallel. The epipolar lines on the two images then lie on the corresponding horizontal lines. Suppose the world coordinates of the spatial point P are $P_{w}$; its projections onto the two camera coordinate systems are $P_{\mathrm{cl}}$ and $P_{\mathrm{cr}}$, respectively. The extrinsic parameters of the left and right cameras are $\left(R_{l},t_{l}\right)$ and $\left(R_{r},t_{r}\right)$, respectively. The relationship between the camera coordinate system and the world coordinate system is as follows:

(20) $$\left\{\begin{array}{c} P_{cl}=R_{l}P_{W}+t_{l}\\ P_{cr}=R_{r}P_{W}+t_{r} \end{array}\right.$$

Disparity *d* is the horizontal difference of corresponding points on the left and right imaging planes. Its calculation formula is as follows:

(21) $$d=x_{l}-x_{r}$$

According to the geometric relationship, the depth *z* (*Z*-direction coordinate) of the object can be calculated by the following formula:

(22) $$z=\frac{bf}{d}$$

Once the disparity map is calculated, the depth information can be calculated through the conversion relationship between disparity and depth.

The initial three-dimensional coordinates of spatial points are obtained using the aforementioned method. Due to factors such as noise in image feature extraction, matching errors, and limited camera calibration accuracy, these initial positions typically contain certain deviations and cannot directly meet the requirements of high-precision localization. To further improve the accuracy of the 3D point coordinates, the system introduces the reprojection error as an optimization criterion. The currently estimated 3D spatial point is back-projected onto each original image plane according to the camera projection model, yielding the corresponding reprojected pixel coordinates. These reprojected coordinates are then compared with the observed feature point coordinates detected in the images, and the geometric distance between them in pixel space is defined as the reprojection error. This error intuitively reflects the deviation between the current estimated 3D point and the true values.

On this basis, with the objective of minimizing the global reprojection error, the three-dimensional coordinates of spatial points are iteratively adjusted via a nonlinear optimization algorithm (e.g., the Levenberg–Marquardt algorithm). In each iteration, the algorithm corrects the position of the 3D point according to the error magnitude and gradient direction, driving the reprojected points to gradually approach the ground truth points. After multiple iterations until convergence, highly accurate and consistent 3D coordinates are finally obtained, which significantly improves the reliability of 3D reconstruction or localization results.

### 2.3. Analysis of the Measurement Method for Alignment of the Rod and Hole

The previous section introduced the conversion principle between image coordinates and spatial coordinates. After image processing to eliminate interference factors such as lighting, followed by edge extraction and corner fitting, the image coordinates of key points on rods and holes can be obtained. The spatial coordinates of key points of holes can be obtained using the corresponding image coordinates [23,24,25,26].

**Figure 5:**
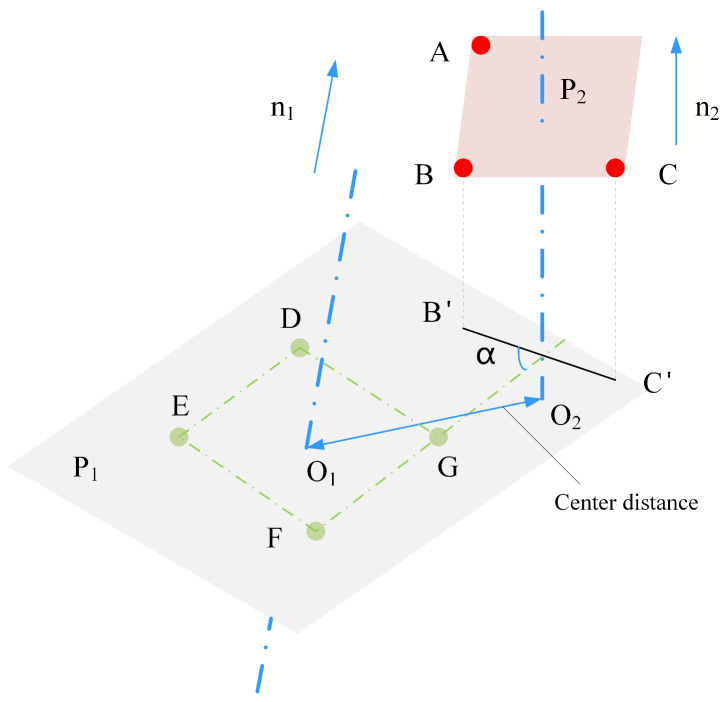
The alignment principle of rod and hole.

Since the relative position of the rods and the camera is fixed, rod–hole alignment mainly involves calculating the relative posture between rods and holes. Therefore, it is necessary to construct an alignment analysis model for rod–hole pairing. As shown in Figure 5, this figure shows the spatial attitude diagram of rods and holes during alignment. The green dots represent the four key points to be measured on the hole. The plane fitted by these four key points is $P_{1}$, and the normal vector of $P_{1}$ is $n_{1}$. The plane fitted by three points on the rod is $P_{2}$, and the normal vector of $P_{2}$ is $n_{2}$. The blue dotted lines represent the central axes of the two planes. The intersection points of the axes of the hole and the rod on plane $P_{1}$ are $O_{1}$ and $O_{2}$, respectively. The distance between $O_{1}$ and $O_{2}$ is the center distance. The projections of points B and C onto plane $P_{1}$ are $B^{\prime }$ and $C^{\prime }$, respectively. Through the control system, plane $P_{2}$ can be adjusted to be parallel to plane $P_{1}$. The angle between line FG and line $B^{\prime }C^{\prime }$ on plane $P_{1}$ is α, which is the rotation angle to be calculated.

The specific 4 steps for aligning rod and hole are as follows:

- Given the position of the camera, the coordinates of the key points A, B, and C on the rods are obtained by binocular vision. Then, the equation of plane $P_{2}$ and the equation of the central axis are fitted according to the key points.
  Given $A=(x_{A},y_{A},z_{A})$, $B=(x_{B},y_{B},z_{B})$, $C=(x_{C},y_{C},z_{C})$, where A, B, and C lie on a square cross-section of a rod, with B being the right-angled vertex, the following formula should theoretically hold:
  (23) $$\left\{\begin{array}{c} \vec{BA}\cdot \vec{BC}=0\\ ∥\vec{BA}∥=∥\vec{BC}∥ \end{array}\right.$$
  The normal vector calculation formula for plane $P_{2}$ is as follows:
  (24) $${\vec{n}}_{2}=\vec{BA}\times \vec{BC}=\left(A-B\right)\times \left(C-B\right)$$
  Normalize the above vector:
  (25) $$n_{2}=\frac{{\vec{n}}_{2}}{∥{\vec{n}}_{2}∥}=\left(a_{2},b_{2},c_{2}\right)$$
  The axis of the rod passes through $O_{s}$ and is perpendicular to the cross-section $P_{2}$, with a direction of $n_{2}$. The equation for the axis is as follows:
  (26) $$r\left(t\right)=O_{s}+t\;n_{2},\;t\in R$$
  The above equation can be rewritten as a symmetric equation:
  (27) $$\frac{x-x_{O_{s}}}{a_{2}}=\frac{y-y_{O_{s}}}{b_{2}}=\frac{z-z_{O_{s}}}{c_{2}}$$
- The least square method is adopted to perform planar fitting (plane $P_{1}$) on the four key points (D, E, F, and G) of the hole, and the coordinates of projection points of the key points on the plane $P_{1}$ are calculated. Connect four projection points to obtain the spatial contour of the hole.
  Given that points D, E, F and G are theoretically coplanar and have square vertices, $D=\left(x_{D},y_{D},z_{D}\right),E=\left(x_{E},y_{E},z_{E}\right),F=\left(x_{F},y_{F},z_{F}\right),G=\left(x_{G},y_{G},z_{G}\right)$. Using the least squares method to fit plane $P_{1}$, the coordinates are centered as shown in the following equation:
  (28) $$\left\{\begin{array}{c} {\bar{P}}_{1}=\left({\bar{x}}_{1},{\bar{y}}_{1},{\bar{z}}_{1}\right)=\frac{D+E+F+G}{4}\\ Q_{i}=P_{i}-{\bar{P}}_{1},\;i\in \left\{D,E,F,G\right\} \end{array}\right.$$
  Construct the covariance matrix as follows:
  (29) $$M_{1}=\sum_{i}Q_{i}Q_{i}^{T}=\left[\begin{array}{ccc} S_{xx} & S_{xy} & S_{xz}\\ S_{xy} & S_{yy} & S_{yz}\\ S_{xz} & S_{yz} & S_{zz} \end{array}\right]$$
  where $S_{xx}=\sum {\left(x_{i}-{\bar{x}}_{1}\right)}^{2},\;S_{xy}=\sum \left(x_{i}-{\bar{x}}_{1}\right)\left(y_{i}-{\bar{y}}_{1}\right).$
  Let $\lambda_{min}$ be the minimum eigenvalue of $M_{1}$, and the corresponding unit eigenvector $n_{1}=\left(a_{1},b_{1},c_{1}\right)$ is the unit normal vector of plane $P_{1}$. The equation for plane $P_{1}$ is as follows:
  (30) $$\left\{\begin{array}{c} a_{1}x+b_{1}y+c_{1}z+d_{1}=0\\ d_{1}=-\left(a_{1}{\tilde{x}}_{1}+b_{1}{\tilde{y}}_{1}+c_{1}{\tilde{z}}_{1}\right) \end{array}\right.$$
  For any point $P=(x_{0},y_{0},z_{0})$, the projected $P^{\prime }$ coordinates on plane $P_{1}$ are as follows:
  (31) $$\left\{\begin{array}{c} t=a_{1}x_{0}+b_{1}y_{0}+c_{1}z_{0}+d_{1}\\ P^{\prime }=\left(x_{0}-a_{1}t,\;y_{0}-b_{1}t,\;z_{0}-c_{1}t\right) \end{array}\right.$$
  By calculating D, E, F, and G separately, $D^{\prime },E^{\prime },F^{\prime }\mathrm{and}\;G^{\prime }$ can be obtained. The sequential connection of points on the hole ($D^{\prime }-E^{\prime }-F^{\prime }-G^{\prime }-D^{\prime }$) results in the spatial quadrilateral contour located on $P_{1}$.
- Calculate the coordinates of the vertical foot $O_{2}$, and take the distance between $O_{2}$ and the $O_{1}$ as the center distance. At the same time, calculate the angle α between the normal vector of the rod and hole plane.
  The formula for solving the intersection point (perpendicular foot $O_{2}$) between the central axis $r\left(t\right)=O_{s}+tn_{2}$ and plane $P_{1}$ is as follows:
  (32) $$\left\{\begin{array}{c} a_{1}\left(x_{O_{s}}+ta_{2}\right)+b_{1}\left(y_{O_{s}}+tb_{2}\right)+c_{1}\left(z_{O_{s}}+tc_{2}\right)+d_{1}=0\\ t_{0}=-\frac{a_{1}x_{O_{s}}+b_{1}y_{O_{s}}+c_{1}z_{O_{s}}+d_{1}}{a_{1}a_{2}+b_{1}b_{2}+c_{1}c_{2}}\\ O_{2}=O_{s}+t_{0}n_{2} \end{array}\right.$$
  The formula for solving the center $O_{1}$ of the hole is as follows:
  (33) $$O_{1}=\frac{D^{\prime }+E^{\prime }+F^{\prime }+G^{\prime }}{4}$$
  The formula for calculating the center distance is as follows:
  (34) $$\Delta =∥O_{1}-O_{2}∥$$
  Projection B and C onto plane $P_{1}$ yields $B^{\prime }$ and $C^{\prime }$, which are calculated using the following formula:
  (35) $$\left\{\begin{array}{c} t_{B}=a_{1}x_{B}+b_{1}y_{B}+c_{1}z_{B}+d_{1}\\ B^{\prime }=\left(x_{B}-a_{1}t_{B},\;-b_{1}t_{B},\;z_{B}-c_{1}t_{B}\right) \end{array}\right.$$
  Similarly, $C^{\prime }$ can be obtained.
  The orientation quantities $\vec{u}=G^{\prime }-F^{\prime }$, $\vec{v}=C^{\prime }-B^{\prime }$, both vectors, are on plane $P_{1}$. Normalize the vector coordinates using the following formula:
  (36) $$\left\{\begin{array}{c} \hat{u}=\frac{\vec{u}}{∥\vec{u}∥}\\ \hat{v}=\frac{\vec{v}}{∥\vec{v}∥} \end{array}\right.$$
  Calculate the angle α according to the dot product formula:
  (37) $$\left\{\begin{array}{c} \cos\alpha =\hat{u}\cdot \hat{v}\\ \alpha =\arccos(\cos\alpha )\in [0,\pi ] \end{array}\right.$$
- Adjust the spatial posture of the lifting gear based on the center distance $O_{1}O_{2}$ and rotation angle α.

## 3. Experiment

### 3.1. Experiment Design

**Figure 6:**
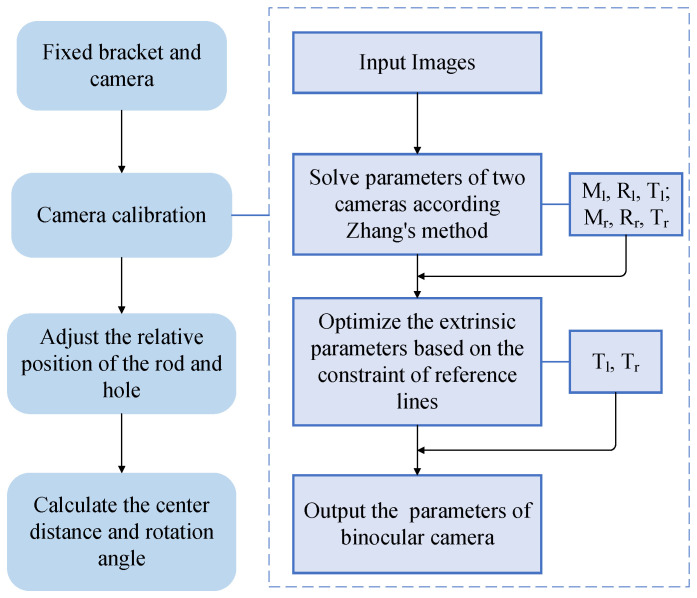
Experiment flow.

The alignment experiment can be divided into three main stages: camera selection, image acquisition, and algorithm accuracy evaluation. The camera should have a suitable frame rate to meet the requirements of hoisting operations and be capable of monitoring the operating status in real time. The deployment position of the camera should be consistent with the actual scene, ensuring imaging quality without interfering with the hoisting process. To ensure the reliability of the experimental procedure, it is necessary to adjust the relative positions of holes and rods during testing. The experimental flow is shown in Figure 6.

**Figure 7:**
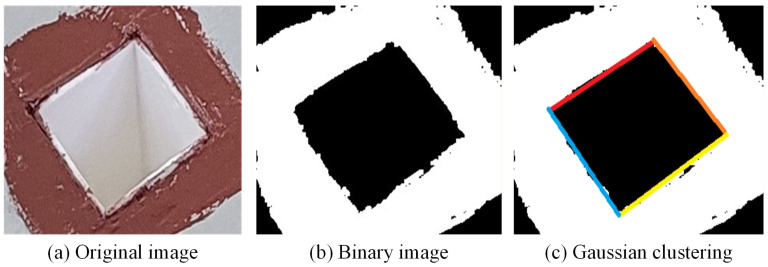
Image process.

The image processing workflow is illustrated in the following figures: Figure 7a presents the original image segmented by the YOLO model. Based on the RGB (Red–Green–Blue) features of the segmented image, binarization is applied, and the corresponding result is shown in Figure 7b. After extracting the inner edges from Figure 7b, the Gaussian mixture clustering algorithm is employed to automatically cluster the edges [27,28,29], thereby obtaining discrete point sets corresponding to the four edges. Subsequently, four straight lines are fitted using these discrete points, and their intersection points are identified as the four corner points of the hole; the result is depicted in Figure 7c, where the four edges are distinguished by four different colors. Once the corner points are determined, corresponding conjugate points are searched for in the other image to obtain stable point pairs.

### 3.2. Construction of the Experimental Platform

**Figure 8:**
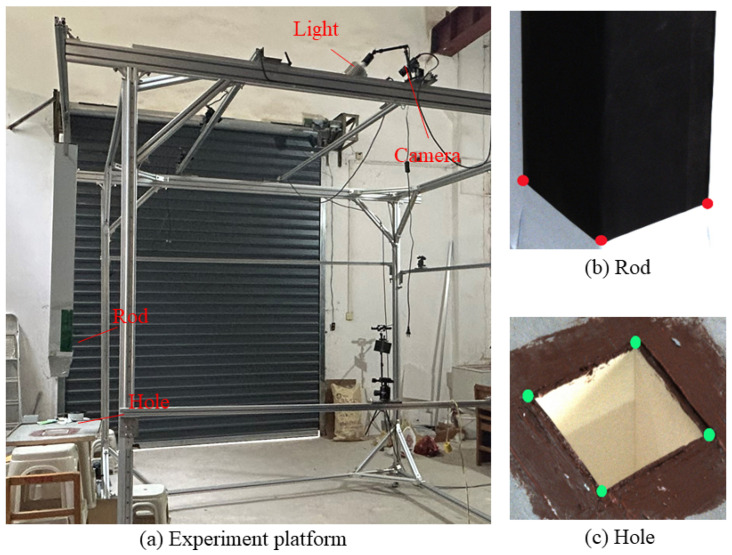
Experiment scenario.

As shown in Figure 8a, in order to more realistically reproduce the scene of the construction site, following the design requirements of the lifting gear and the dimensions of the segmented beams, a three-dimensional model of the segmented beams hoisting operation is established with a total of six rods and six holes. To verify the proposed method, an experimental platform required for visual hoisting is built according to the actual design scale to simulate the working states of individual rods and holes. A stereo camera is installed on the stand. The position and angle of the camera can be adjusted to complete the shooting at the best angle and distance. To ensure clear imaging, a light source is installed on the bracket.

To address the core technical challenge of optimal deployment of visual monitoring cameras in large-scale construction areas, as well as the significant challenges posed by the complex construction site environment and variable natural lighting conditions on camera calibration and image acquisition, this paper, in combination with practical engineering needs, carried out camera installation position optimization and lighting environment adaptation design, and it rigorously verified the feasibility and rationality of the solution through experiments. Considering the actual structural form, spatial dimensions, and operational conditions of the engineering lifting equipment, a comprehensive analysis of key factors such as the camera’s field of view coverage, target image clarity, installation stability, and ease of subsequent maintenance was conducted to determine the optimal installation positions of the cameras. In the experimental platform, the arrangement positions and installation attitudes of the cameras are completely consistent with the installation state in actual engineering projects, ensuring a high degree of match between the experimental environment and real construction scenarios, and guaranteeing that the experimental results have direct engineering reference value and transferability.

To effectively mitigate the adverse effects of uneven lighting, strong light interference, and shadow changes at the construction site on image quality, and to improve visual recognition and positioning accuracy, a dedicated auxiliary lighting source is added to the experimental system. The type, power, arrangement, and angle of the light are consistent with the lighting configuration on the actual hoist, maximally restoring the real working environment, effectively reducing the interference of lighting changes on visual measurement accuracy, and enhancing the system’s robustness under complex site conditions. In terms of camera spatial position parameter design, a refined quantitative optimization was carried out: the distance between the camera and the hoist in the Y direction is set to 0.5 m, which ensures the complete imaging of the target while taking into account structural interference risks and compact installation; considering the constraints on the relative distance of the hoist holes from the preliminary positioning of the previous stage of laser ranging, that is, the hole distance needs to be maintained within the range of 0.2 m to 0.3 m, and by balancing positioning accuracy with spatial layout, the total distance in the Z direction is set to 1.85 m in the experiment; fully considering multiple constraints such as the camera’s field of view, imaging resolution, depth of field requirements, hoist structure strength, and motion stability, after multiple sets of simulations and preliminary experiments for comparative analysis, the final relative distance between the camera and the hoist in the X direction is set to 2.2 m, enabling the visual system to stably acquire high-quality images throughout the entire lifting process, and meeting the accuracy requirements of visual positioning and intelligent hoisting.

The above camera positioning optimization scheme and lighting compensation strategy effectively address practical engineering problems such as the difficulty of camera deployment in large-scale construction areas, the difficulty of on-site calibration, and poor lighting adaptability, demonstrating not only the theoretical rationality of the proposed method but also fully validating its practical application value and promotion prospects in real engineering projects.

As shown in Figure 8b,c, the three red marked points on the rods are the points to be measured, and the four green marked points on the hole are the points to be measured. The relative pose of the rod and hole is measured by these seven points.

As shown in Table 1, these are the intrinsic parameters of the stereo camera. The pixel size is 3.45 μm, with $u_{x}$ and $u_{y}$ being the coordinates of the main image points and $k_{1}$ and $k_{2}$ being the radial distortion parameters of the camera, and the image resolution is 4096 × 3000.

The extrinsic parameters of the camera are shown in Table 2. *X*, *Y* and *Z* represent the coordinates of the camera’s projection center, and φ, ω, and κ are the rotation angles of the image in the three directions.

**Figure 9:**
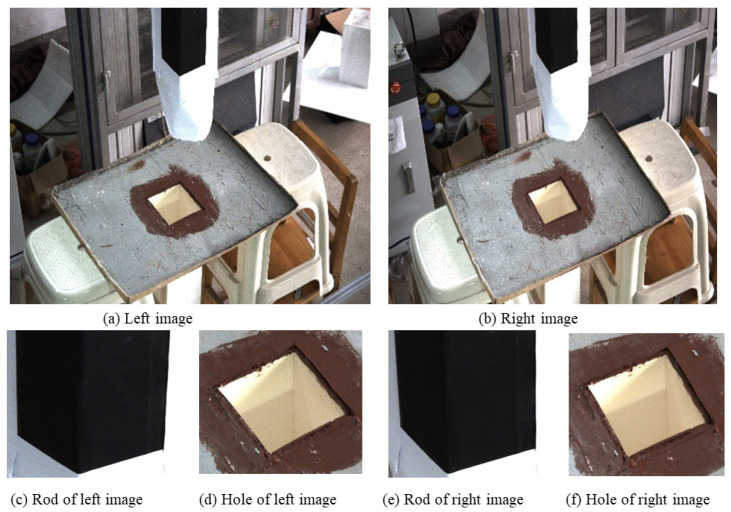
Real photos.

Figure 9a,b presents an image captured by a stereo camera. Both the left and right images contain a rod and a hole. The rod and hole are separated by using an image segmentation algorithm, and image processing is carried out. The processed local image is shown in Figure 9c–f. The main features of the rod and hole are retained on the local image, and the parts unrelated to the rod and hole are removed. Image processing is carried out on the local image to obtain the image coordinates of key points in the left image, and the epipolar constraint is used to match the image coordinates of key points in the right image. According to the principle of binocular vision, the image coordinates are converted into spatial coordinates, which facilitates the calculation of the relative positions of the hole and rod.

To fully verify the measurement accuracy and robustness of the proposed method, 30 independent experiments were conducted by adjusting the spatial position of the hoisting hole to vary the relative pose and distance between the suspender and the hoisting hole while keeping the positions of the suspender and the camera unchanged. The measurement results from a total station were adopted as the ground truth of spatial coordinates against which the measurement results of the proposed method were compared. Meanwhile, the standard deviation and root mean square error (RMSE) of the rotation angle and center distance were calculated, and the confidence intervals were derived at the 95% confidence level. The detailed experimental data and error analysis are presented in Table 3 and Table 4.

Based on the experimental data and error statistics from 30 independent tests, the proposed measurement method achieves high accuracy and favorable stability.

For rotation angle measurement, the absolute error ranges from 0.06° to 0.45°, and the relative error ranges from 0.73% to 6.21% with most values below 3%. The standard deviation is 0.123°, the RMSE is 0.215°, and the 95% confidence interval is [0.172, 0.258], indicating small error dispersion and high reliability.

For center distance measurement, the absolute error ranges from 0.14 mm to 1.73 mm, and the relative error ranges from 0.26% to 1.57%. The standard deviation is 0.452 mm, the RMSE is 0.683 mm, and the 95% confidence interval is [0.561, 0.805], confirming stable measurements that meet practical engineering accuracy requirements.

Overall, the proposed method achieves high-precision measurement under varying hole positions and relative poses, validating its robustness and engineering applicability.

This experimental system establishes a complete technical process integrating image acquisition, visual processing, mathematical modeling, and data analysis. The system’s stability is verified through multiple sets of repeatability experiments. The experimental results indicate that the deviations in the rotation angles of the hoist hole and hoist rod, as well as the deviation in the center distance, are all within the allowable engineering error range. The overall measurement accuracy is high, and it is capable of meeting the technical specifications and usage requirements of practical engineering applications.

## 4. Conclusions

To improve the safety and efficiency of rod–hole alignment during precast beam hoisting, this paper proposes a binocular vision-based visual alignment method for precast beam hoisting. This method adopts a stereo camera to acquire images of rods and holes, extracts image key points, and matches these points via epipolar geometry to calculate the spatial coordinates of key points on rods and holes. According to the spatial positional relationship of these key points, a mathematical model is established to compute the center distance and rotation angle between rods and holes.

To validate the practical performance of the proposed method, an experimental platform for rod–hole alignment is constructed based on real engineering scenarios. The positions of rods and the camera remain fixed, while the relative pose between rods and holes is adjusted by moving holes. Experimental results show that the maximum absolute error of center distance is less than 1.73 mm, and the maximum absolute error of rotation angle is merely 0.47°. Both indicators fully meet practical engineering requirements.

The established experimental platform only completes alignment tests for a single rod and hole pair. Practical construction requires the simultaneous alignment of six pairs of rods and holes. Moreover, the current test platform still differs from actual working conditions. Accordingly, future research will focus on conducting alignment experiments for six pairs of rods and holes under real engineering environments [30,31,32,33].

## Figures and Tables

**Table 1:** Intrinsic parameters of camera.

Parameters	Cam1	Cam2
*f* (μm)	3.455	3.449
$u_{x}$ (pixel)	2070.56	2070.79
$u_{y}$ (pixel)	1514.27	1501.06
$k_{1}$	0.0121	−0.0508
$k_{2}$	−1.0502	0.9882
width (pixel)	4096	4096
height (pixel)	3000	3000

**Table 2:** Extrinsic parameters of camera.

	*X*	*Y*	*Z*	φ (rad)	ω (rad)	κ (rad)
Cam1	468.95	27.97	2530.64	6.13	3.30	1.91
Cam2	424.31	327.52	2437.24	6.16	3.17	1.83

**Table 3:** Alignment results of rod and hole.

Number	Index	Proposed	True	Absolute Error	Relative Error
1	Rotation (°)	6.92	6.85	0.07	1.02%
	Distance (mm)	54.29	53.87	0.42	0.78%
2	Rotation (°)	6.29	6.39	0.10	1.57%
	Distance (mm)	54.31	54.45	0.14	0.26%
3	Rotation (°)	7.67	7.36	0.31	4.21%
	Distance (mm)	103.47	104.90	1.43	1.36%
4	Rotation (°)	15.53	15.98	0.45	2.82%
	Distance (mm)	108.45	110.18	1.73	1.57%
5	Rotation (°)	4.68	4.99	0.31	6.21%
	Distance (mm)	19.54	19.38	0.16	0.83%
6	Rotation (°)	8.28	8.22	0.06	0.73%
	Distance (mm)	72.40	72.15	0.25	0.35%
7	Rotation (°)	10.12	10.35	0.23	2.22%
	Distance (mm)	88.62	89.05	0.43	0.48%
8	Rotation (°)	5.78	5.92	0.14	2.37%
	Distance (mm)	36.89	36.72	0.17	0.46%
9	Rotation (°)	12.35	12.18	0.17	1.39%
	Distance (mm)	95.76	96.92	1.16	1.20%
10	Rotation (°)	7.89	7.76	0.13	1.67%
	Distance (mm)	65.32	65.58	0.26	0.39%
11	Rotation (°)	14.21	14.53	0.32	2.20%
	Distance (mm)	115.47	116.89	1.42	1.21%
12	Rotation (°)	5.23	5.38	0.15	2.79%
	Distance (mm)	28.65	28.49	0.16	0.56%
13	Rotation (°)	9.45	9.28	0.17	1.83%
	Distance (mm)	82.13	82.56	0.43	0.52%
14	Rotation (°)	11.76	11.92	0.16	1.34%
	Distance (mm)	99.87	100.32	0.45	0.45%
15	Rotation (°)	6.54	6.67	0.13	1.95%
	Distance (mm)	49.28	49.05	0.23	0.47%
16	Rotation (°)	13.58	13.89	0.31	2.23%
	Distance (mm)	107.54	108.96	1.42	1.30%
17	Rotation (°)	5.89	6.05	0.16	2.64%
	Distance (mm)	39.76	39.58	0.18	0.45%
18	Rotation (°)	8.76	8.62	0.14	1.62%
	Distance (mm)	76.32	76.68	0.36	0.47%
19	Rotation (°)	10.89	11.12	0.23	2.07%
	Distance (mm)	89.45	89.92	0.47	0.52%
20	Rotation (°)	7.23	7.15	0.08	1.12%
	Distance (mm)	58.76	58.59	0.17	0.29%
21	Rotation (°)	12.98	13.25	0.27	2.04%
	Distance (mm)	102.34	103.76	1.42	1.37%
22	Rotation (°)	5.45	5.62	0.17	3.02%
	Distance (mm)	32.18	32.03	0.15	0.47%
23	Rotation (°)	9.87	9.73	0.14	1.44%
	Distance (mm)	85.67	86.02	0.35	0.41%
24	Rotation (°)	14.76	15.12	0.36	2.38%
	Distance (mm)	112.89	114.25	1.36	1.19%
25	Rotation (°)	6.89	7.02	0.13	1.85%
	Distance (mm)	52.45	52.28	0.17	0.33%
26	Rotation (°)	8.34	8.21	0.13	1.58%
	Distance (mm)	70.12	70.45	0.33	0.47%
27	Rotation (°)	11.23	11.45	0.22	1.92%
	Distance (mm)	92.78	93.21	0.43	0.46%
28	Rotation (°)	5.12	5.27	0.15	2.85%
	Distance (mm)	26.89	26.74	0.15	0.56%
29	Rotation (°)	13.12	13.45	0.33	2.45%
	Distance (mm)	105.67	107.02	1.35	1.26%
30	Rotation (°)	9.15	9.08	0.07	0.77%
	Distance (mm)	78.62	78.95	0.33	0.42%

**Table 4:** Error statistics and confidence interval analysis (95% confidence level).

Evaluation Index	Standard Deviation	RMSE	Confidence Interval
Rotation (°)	0.123	0.215	[0.172, 0.258]
Distance (mm)	0.452	0.683	[0.561, 0.805]

## Data Availability

The datasets used and/or analyzed during the current study are available from the corresponding author upon reasonable request.
